# Amphiphilic Poly(N-vinylpyrrolidone) Biocomposites with Bortezomib and DR5-Selective TRAIL Variants: A Promising Approach to Pancreatic Cancer Treatment

**DOI:** 10.3390/ijms262311620

**Published:** 2025-11-30

**Authors:** Ekaterina Kukovyakina, Alina A. Isakova, Dmitry Bagrov, Marine Gasparian, Andrey Kuskov, Anne Yagolovich

**Affiliations:** 1 Department of Technology of Chemical, Pharmaceutical and Cosmetic Substances, D. Mendeleev University of Chemical Technology of Russia, 125047 Moscow, Russia; kukoviakina.e.v@muctr.ru (E.K.); alina.labbio@gmail.com (A.A.I.); kuskov.a.n@muctr.ru (A.K.); 2 Faculty of Biology, Lomonosov Moscow State University, 119234 Moscow, Russia; bagrov@mail.bio.msu.ru; 3 Shemyakin-Ovchinnikov Institute of Bioorganic Chemistry of the Russian Academy of Sciences, 117997 Moscow, Russia

**Keywords:** bortezomib, TRAIL, DR5 receptor, iRGD peptide, amphiphilic polymer N-vinylpyrrolidone, biocomposite, pancreatic cancer

## Abstract

A promising strategy for pancreatic cancer therapy involves developing nanocarriers capable of simultaneously delivering various antitumor substances with diverse physicochemical properties, often resulting in synergistic effects. In the present work, novel biocomposites were developed using amphiphilic N-vinylpyrrolidone polymer incorporating bortezomib (BTZ) and modified with either the DR5-selective TRAIL cytokine (DR5-B) or its fusion with the iRGD effector peptide (DR5-B-iRGD), resulting in AmphPVP-BTZ-DR5-B and AmphPVP-BTZ-DR5-B-iRGD formulations. The release of BTZ was most extensive at acidic pH 5.6, mimicking endolysosomal compartments, while at near-neutral pH 7.4 and alkaline pH 8.2 the release was slower and less complete, indicating a smart pH-responsive behavior suitable for triggered release in the tumor microenvironment. Both AmphPVP-BTZ-DR5-B and AmphPVP-BTZ-DR5-B-iRGD significantly inhibited the growth of pancreatic adenocarcinoma cell lines PANC-1, BxPC-3, and MIA PaCa-2 and induced more rapid internalization of the DR5 receptor in MIA PaCa-2 cells than unmodified particles and free DR5-B or DR5-B-iRGD. Importantly, AmphPVP-BTZ-DR5-B-iRGD exhibited a more pronounced DR5 internalization rate and cytotoxic effect than AmphPVP-BTZ-DR5-B owing to the presence of fusion protein with internalizing iRGD peptide. Both biocomposites induced cell death via the apoptotic pathway while exhibiting minimal cytotoxic effects on healthy cells. Therefore, biocomposites incorporating BTZ and functionalized with DR5-selective TRAIL variants DR5-B or DR5-B-iRGD represent a promising avenue for future studies in pancreatic cancer animal models.

## 1. Introduction

Pancreatic cancer is a highly lethal malignancy with a significant mortality rate worldwide [1,2]. It is typically diagnosed at advanced stages, exhibits aggressive behavior, and rapidly forms metastases. In addition, pancreatic cancer exhibits substantial resistance to most conventional treatments, including chemotherapy, radiation therapy, and molecular targeted therapy [3]. Although gemcitabine has been the standard first-line chemotherapy for pancreatic cancer since 1997, prolonged treatment leads to chemoresistance in patients [4]. Therefore, the search for new drug molecules is an urgent task for the treatment of pancreatic cancer. Hence, the discovery of novel therapeutic agents represents an urgent priority in pancreatic cancer treatment.

The proteasome inhibitor bortezomib (BTZ) has been approved by the US Food and Drug Administration for the treatment of hematological malignancies [5]. Currently, bortezomib is being evaluated in clinical trials for various cancer types, including pancreatic cancer [6,7]. BTZ has been proven to trigger apoptosis in pancreatic cancer cells [8,9]. Nevertheless, BTZ exhibits toxicity to healthy organs and tissues, accompanied by several adverse effects, such as peripheral neuropathy and thrombocytopenia [10,11].

To mitigate side effects, BTZ is encapsulated within various nanocarriers, including liposomes, dendrimers, polymer micelles and nanogels [12,13,14,15,16]. Among the wide range of nanocarriers, polymer carriers formed by self-aggregation into “hydrophobic core-hydrophilic shell” nanostructures in aqueous media have gained wide application in drug delivery [17]. These carriers are composed of block copolymers containing hydrophobic and hydrophilic segments or polymers with amphiphilic structures [18,19,20,21,22]. The advantages of polymeric nanocarriers include prolonged drug release, ease of surface modification, a wide range of therapeutic agent loading capabilities, as well as biocompatibility and low toxicity [23,24,25]. For example, amphiphilic poly-N-vinylpyrrolidone aggregates are able to effectively immobilize low molecular weight biologically active substances and protein compounds, incorporate into liposomal membrane structures, and maintain biocompatibility during interactions with blood components [26,27,28,29,30].

For efficient tumor targeting, the carrier surface is functionalized with various ligands, including antibodies, aptamers, peptides, carbohydrates, or folic acid [31,32,33,34]. Among promising ligands, TRAIL (TNF-Related Apoptosis-Inducing Ligand) stands out due to its selective interaction with DR4 or DR5 death receptors, triggering apoptosis in malignant cells without affecting healthy cells [35]. Considering that DR5 is recognized as the key receptor in TRAIL-mediated apoptosis signaling, a receptor-selective TRAIL variant DR5-B with enhanced DR5 selectivity was developed to improve antitumor activity [36]. To further enhance its efficacy, a bispecific fusion protein DR5-B-iRGD containing an iRGD peptide was created, exhibiting improved permeability through tumor tissues [37].

The concurrent delivery of two or more antitumor agents represents a promising strategy in cancer treatment allowing for the simultaneous targeting of various mechanisms involved in tumor growth and development while reducing the required dosage of therapeutic substances and minimizing the risk of drug resistance [38,39,40]. The present study focused on the fabrication of the biocomposites based on the amphiphilic polymer N-vinylpyrrolidone, incorporating BTZ and modified with either DR5-B or DR5-B-iRGD protein. These AmphPVP-BTZ-DR5-B and AmphPVP-BTZ-DR5-B-iRGD biocomposites were expected to demonstrate superior DR5 receptor internalization, efficient triggering of apoptotic cell death, and enhanced cytotoxic effects against pancreatic cancer cell lines. This combinatorial approach is of significant importance as it aims to simultaneously target different apoptotic pathways (proteasome inhibition and death receptor activation), potentially overcoming the resistance mechanisms that often limit the efficacy of single-agent therapies in pancreatic cancer. Comparative evaluation of these formulations will assess the potential of the iRGD peptide to enhance antitumor efficacy in pancreatic cancer cells.

## 2. Results

### 2.1. Fabrication and Characterization of the Biocomposites AmphPVP-BTZ-DR5-B and AmphPVP-BTZ-DR5-B-iRGD

**Figure 1:**
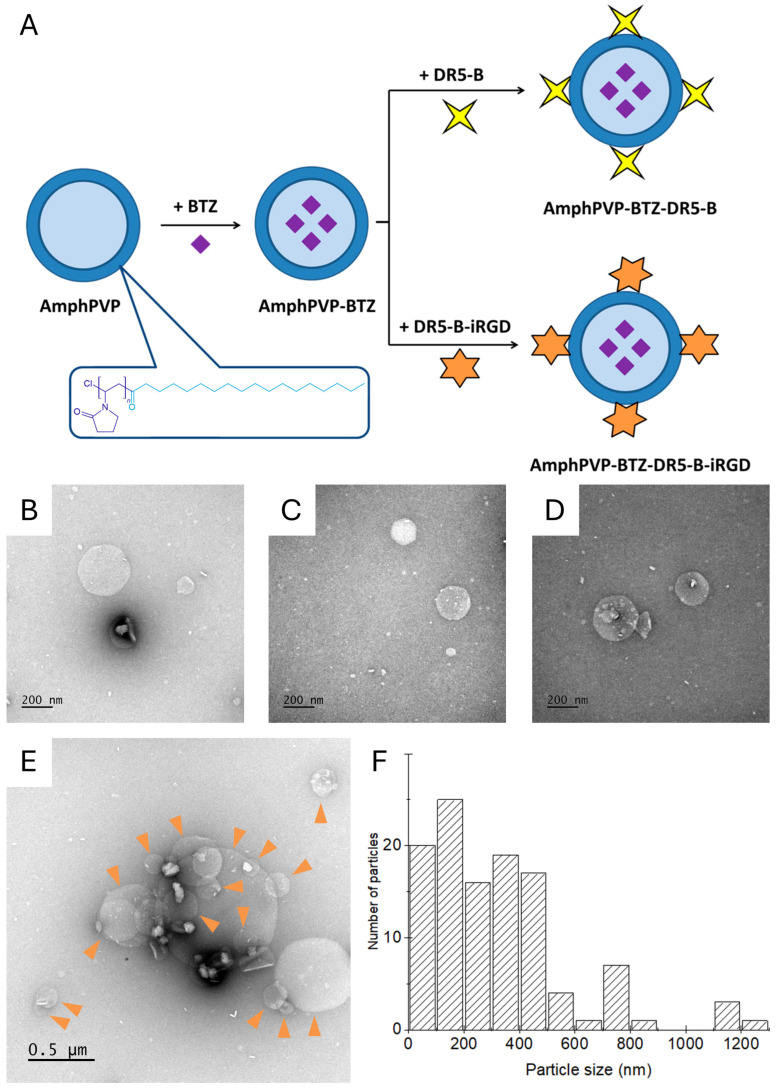
Fabrication and characterization of the biocomposites. (**A**) Scheme of fabrication of AmphPVP-BTZ-DR5-B and AmphPVP-BTZ-DR5-B-iRGD formulations; (**B**–**E**) TEM images of the AmphPVP-BTZ-DR5-B particles, with individual particles marked by triangles in (**E**); (**F**) the size distribution histogram (sample size *N* = 114).

The AmphPVP-based biocomposites were obtained from a synthetic amphiphilic poly(N-vinylpyrrolidone) polymer with one terminal hydrophobic stearoyl group and a molecular weight of 11 kDa. The synthesis and comprehensive characterization of this polymer, including its critical aggregation concentration (CAC ~ 9.7 μmol/L) and structural analysis, have been extensively described in our previous publications [41]. For the present study, AmphPVP was used as a standardized reagent from a well-characterized batch. This amphiphilic polymer at concentrations over CAC in aqueous media forms stable spherical aggregates, with the stearoyl group forming the hydrophobic core and poly(N-vinylpyrrolidone) constituting the hydrophilic shell, having an average size of about 150–180 nm, polydispersity index (PDI) of about 0.15, and zeta-potential of about −8 mV [41]. In aqueous media, the AmphPVP self-assembles into nano-sized aggregates, with the stearoyl group forming the hydrophobic core and poly(N-vinylpyrrolidone) constituting the hydrophilic shell. Then, a hydrophobic low-molecular-weight BTZ was incorporated into polymeric particles with loading efficiency of about 99.8% and loading capacity of 1.5%, followed by modification with a targeted ligand, the DR5-selective variant cytokine TRAIL (DR5-B) or its fusion protein DR5-B-iRGD (Figure 1A). Thus, AmphPVP-BTZ-DR5-B and AmphPVP-BTZ-DR5-B-iRGD biocomposites were successfully fabricated.

The biocomposites were visualized using a transmission electron microscope (Figure 1B–E). They appeared round shaped, with subtle structural inhomogeneities, in agreement with the previous data on similar particles [42,43]. The mean diameter was 320 ± 250 nm (mean ± standard deviation), and the size distribution histogram is shown in Figure 1F. The z-average size, surface charge, and polydispersity index were measured using dynamic light scattering (DLS). The main characteristics of biocomposites are shown in Table 1. The differences in size observed by TEM (320 nm) and by DLS (680 nm) may be attributed to the relatively high PDI and the overall positive skewness of the particle size distribution [44]. Indeed, DLS is usually highly sensitive to the presence of the large particles in the sample, and it can be the reason for the discrepancy between the z-average diameter measured using DLS and the mean size measured using TEM. Furthermore, the DLS measurement evaluates the hydrodynamic diameter of the solvated particles, including the hydrophilic PVP corona, whereas TEM visualizes the particles in a dry, collapsed state, which typically results in a smaller apparent size. The intensity-weighted DLS distributions for all formulations, provided in Supplementary Figure S1, were monomodal, confirming a single population of particles. The zeta potential of the plain AmphPVP particles became less negative upon loading with BTZ and further upon modification with the proteins (Table 1), consistent with the incorporation of the drug and the adsorption of the protein ligands onto the particle surface.

### 2.2. In Vitro Release of Either DR5-B or DR5-B-iRGD and BTZ from the Biocomposites

**Figure 2:**
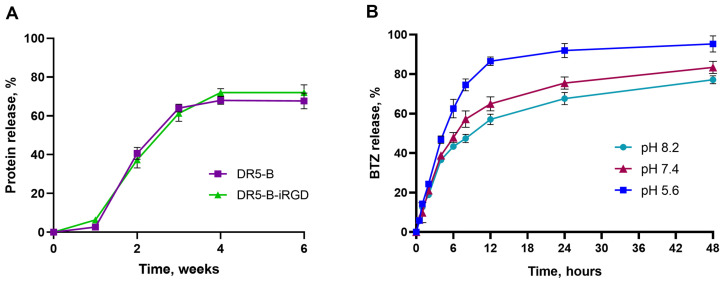
Drug release profiles from the biocomposites: (**A**) Release of DR5-B or DR5-B-iRGD from AmphPVP-BTZ-DR5-B or AmphPVP-BTZ-DR5-B-iRGD, respectively; (**B**) Release of BTZ from biocomposites at pH 5.6, 7.4, and 8.2.

The in vitro release profiles of DR5-B or DR5-B-iRGD from the AmphPVP-BTZ nanoparticles were studied during 6 weeks (Figure 2A). Both proteins demonstrated similar release profiles. Extensive release (from 5% to 40%) was observed between the 1st and 2nd week post fabrication with a subsequent decrease in release rate. During the 6-week period, about 65% of proteins were released from the biocomposites.

The in vitro release profiles of BTZ from the biocomposites were investigated at pH levels of 5.6, 7.4, and 8.2 over a 48 h period. The results presented in Figure 2B demonstrate a clear and pronounced pH-dependent release kinetic. At acidic pH 5.6, the release was most rapid and extensive, reaching 92.4% after 48 h. Conversely, the release was slower and less complete at physiological pH 7.4 (84.3% at 48 h) and slowest at alkaline pH 8.2 (77.5% at 48 h). The differences were most evident at the 8 h time point, with release percentages of 73.3%, 54.6%, and 48.3% for pH 5.6, 7.4, and 8.2, respectively.

The release data were analyzed using four different kinetic models. The correlation coefficients (R^2^) for all models and the key parameters for the Korsmeyer–Peppas model are summarized in Table 2.

The analysis of the correlation coefficients revealed that the Korsmeyer–Peppas model provided the best fit for the release data at all three pH values (R^2^ > 0.998). The Higuchi model also showed very good results (R^2^ > 0.98), while the first-order and zero-order models provided progressively poorer fits.

The parameters derived from the Korsmeyer–Peppas model were highly informative. The release exponent (n) decreased with increasing pH: 0.67 at pH 5.6, 0.58 at pH 7.4, and 0.52 at pH 8.2. Furthermore, the release rate constant (*K*) followed the same trend, being highest at acidic pH and lowest at alkaline pH.

### 2.3. Assessment of the Cytotoxic Activity of AmphPVP-BTZ-DR5-B and AmphPVP-BTZ-DR5-B-iRGD Biocomposites in Pancreatic Cancer Cell Lines

**Figure 3:**
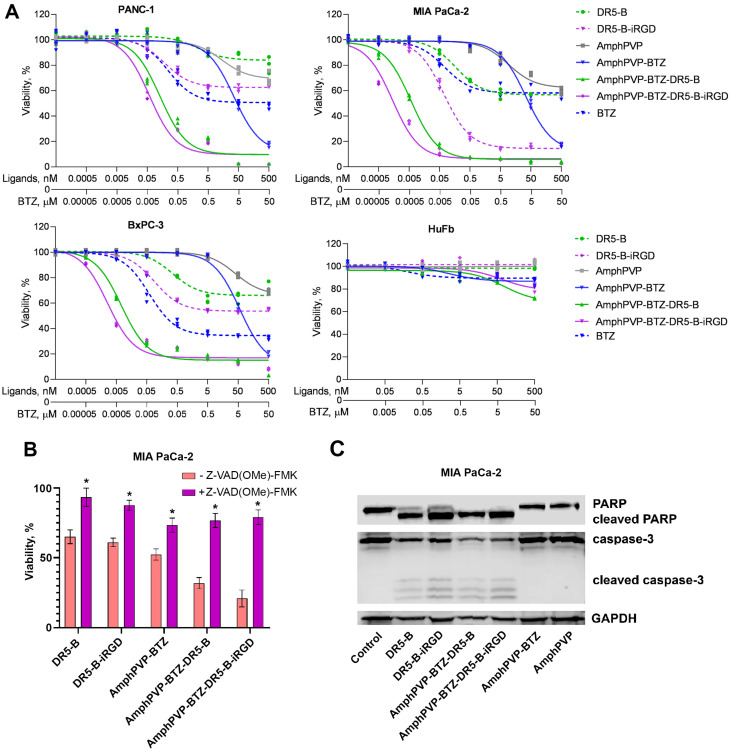
Biological activity of the biocomposites: (**A**) Viability of pancreatic cancer cell lines PANC-1, BxPC-3, and MIA PaCa-2 and normal human fibroblasts (HuFb) evaluated after 24 h incubation with DR5-B, DR5-B-iRGD, AmphPVP, AmphPVP-BTZ, AmphPVP-BTZ-DR5-B, or AmphPVP-BTZ-DR5-B-iRGD; MTT test; (**B**) Cytotoxicity effects of 50 nM of DR5-B, DR5-B-iRGD, AmphPVP-BTZ, AmphPVP-BTZ-DR5-B, or AmphPVP-BTZ-DR5-B-iRGD in MIA PaCa-2 cells after 48 h incubation in the presence of the pan-caspase inhibitor Z-Vad(OMe)-FMK. One-way ANOVA followed by Tukey’s multiple comparisons test, * *p* < 0.05; (**C**) Western blot analysis of PARP and caspase-3 cleavage in MIA PaCa-2 cells induced by 50 nM of ligands after 4 h incubation.

The cytotoxicity of biocomposites was assessed using a panel of pancreatic adenocarcinoma cell lines PANC-1, BxPC-3, and MIA PaCa-2. After 24 h incubation with AmphPVP-BTZ-DR5-B or AmphPVP-BTZ-DR5-B-iRGD, all cell lines showed a significant decrease in viability compared to free proteins DR5-B, DR5-B-iRGD and non-modified AmphPVP-BTZ particles. Notably, both AmphPVP-BTZ-DR5-B and AmphPVP-BTZ-DR5-B-iRGD biocomposites exhibited outstanding cytotoxicity at concentrations where neither DR5-B, DR5-B-iRGD, nor AmphPVP-BTZ exhibited cytotoxic effects. Expectedly, AmphPVP-BTZ-DR5-B-iRGD was more cytotoxic than AmphPVP-BTZ-DR5-B in all studied cancer cell lines due to the presence of cell-penetrating iRGD peptide (Figure 3A). Notably, while none of the individual components (AmphPVP, DR5-B, and DR5-B-iRGD) displayed cytotoxicity toward healthy human fibroblasts HuFb, the BTZ-containing AmphPVP-BTZ, AmphPVP-BTZ-DR5-B, and AmphPVP-BTZ-DR5-B-iRGD biocomposites exhibited slight cytotoxicity at their maximal concentrations, possibly due to BTZ-mediated effects (Figure 3A). Free BTZ reduced cell viability at lower concentrations than BTZ incorporated in AmphPVP BTZ, likely because it does not require release from the AmphPVP particles.

The half maximal inhibitory concentrations (IC_50_) of the studied formulations are represented in Table 3.

It is worth noting that PANC-1, BxPC-3, and MIA PaCa-2 cell lines have different sensitivity to DR5-B. Thus, in DR5-B-resistant PANC-1 cells (survival rate ~90%), the IC_50_ values of AmphPVP-BTZ-DR5-B and AmphPVP-BTZ-DR5-B-iRGD were 0.13 nM and 0.05 nM, correspondingly. In DR5-B-sensitive BxPC3 and MIA PaCa-2 cell lines (survival rates ~70% and ~60%, respectively), the IC_50_ values were 0.007 and 0.006 nM after incubation with AmphPVP-BTZ-DR5-B and 0.003 and 0.001 nM with AmphPVP-BTZ-DR5-B-iRGD, respectively (Table 3). Importantly, the iRGD domain in the DR5-B-iRGD fusion protein improved the cytotoxic efficacy with IC_50_ values reduced by 13-, 3.9-, and 2.4-fold versus DR5-B in PANC-1, BxPC-3, and MIA PaCa-2 cells, respectively. Consistently, AmphPVP-BTZ-DR5-B-iRGD biocomposites exhibited enhanced cytotoxicity relative to AmphPVP-BTZ-DR5-B, showing decreases of IC_50_ values of 2.2-, 2.3-, and 6-fold in PANC-1, BxPC-3, and MIA PaCa-2 cells, respectively.

To reveal the mechanistic evidence of the cytotoxicity of the biocomposites, we have pre-treated MIA PaCa-2 cells with pan-caspase inhibitor Z-Vad(OMe)-FMK. A significant increase in viability was observed in Z-Vad(OMe)-FMK-treated cells, indicating that apoptosis is the main mechanism of AmphPVP-BTZ-DR5-B and AmphPVP-BTZ-DR5-B-iRGD cytotoxic activity in pancreatic cancer cells (Figure 3B). This was supported by Western blot analysis: PARP and caspase-3 were efficiently cleaved in MIA PaCa-2 cells following exposure to Amph PVP BTZ-DR5-B and Amph PVP BTZ-DR5-B-iRGD, as well as to DR5-B and DR5-B-iRGD proteins, indicating activation of the apoptotic pathway (Figure 3C and Figure S2).

### 2.4. DR5 Receptor Internalization by Biocomposites

**Figure 4:**
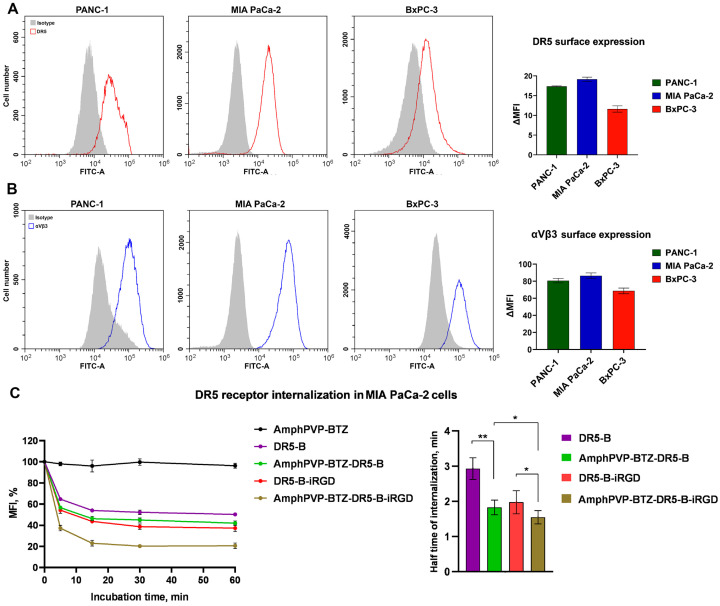
DR5 receptor internalization after exposure to biocomposites: Surface expression levels of (**A**) DR5 and (**B**) integrin αvβ3 receptor in PANC-1, MIA PaCa-2, and BxPC-3 cell lines estimated by flow cytometry; (**C**) DR5 receptor internalization curves in MIA PaCa-2 cells after either 5, 15, 30, or 60 min incubation with 5 nM of DR5-B, DR5-B-iRGD, AmphPVP-BTZ, AmphPVP-BTZ-DR5-B, or AmphPVP-BTZ-DR5-B-iRGD biocomposites measured by flow cytometry, and half-time of internalization calculated via nonlinear regression option in GraphPad Prism 8.0. One-way ANOVA followed by Tukey’s multiple comparisons test, * *p* < 0.05, ** *p* < 0.005.

To elucidate the mechanism laying behind the enhanced cytotoxicity of the biocomposites, their effect on DR5 receptor internalization was investigated. The study was performed in MIA PaCa-2 cells, because they exhibited the highest sensitivity among pancreatic cell lines in viability assays (Figure 3A) and expressed high levels of the DR5 receptor on their surface (Figure 4A). It is well established that binding of both TRAIL and its DR5-selective variant DR5-B triggers rapid internalization of the DR5 receptor [45]. Immobilization of DR5-B on the surface of AmphPVP-BTZ particles resulted in increased DR5 internalization rate, presumably due to the enhanced DR5 receptor clustering. All three studied cancer cell lines abundantly expressed integrin αvβ3, the target of iRGD peptide, on the cell surface (Figure 4B). Consistently, the functionalization of AmphPVP-BTZ with the DR5-B-iRGD fusion protein resulted in a further reduction in DR5 internalization half-time. This effect was observed for AmphPVP-BTZ-DR5-B-iRGD compared to AmphPVP-BTZ-DR5-B as well as for DR5-B-iRGD compared to DR5-B ligand (Figure 4C). While these data strongly support enhanced receptor engagement and clustering, direct visualization of particle internalization using fluorescently labeled composites would provide deeper mechanistic insight in future studies.

## 3. Discussion

Polymer nanocarriers represent a versatile tool for the co-delivery of various antitumor molecules [46,47,48,49] and a promising approach in pancreatic cancer therapy by delivering combinations of antitumor agents that target both tumor cell proliferation and their microenvironment. For example, successful combinations include paclitaxel with phosphorylated gemcitabine [50], miR-let7b with Hh inhibitor GDC-0449 [51], Hh inhibitor cyclopamine with EGFR inhibitor gefitinib [52], and doxorubicin and PI3K inhibitor with phenylboronic acid [53]. TRAIL ligand modification is also commonly employed in the design of polymeric nanoformulations (reviewed in [54]). For example, Nguyen et al. loaded cathepsin K inhibitor odanacatib into poly(lactic-co-glycolic) nanoparticles conjugated to TRAIL fusion protein for targeting TRAIL-resistant cancer [55]. Furthermore, the potential of targeting the DR5 pathway is highlighted by a recent study demonstrating the potent efficacy of an anti-DR5 antibody–drug conjugate (Oba01), which delivers the cytotoxic agent monomethyl auristatin E (MMAE) to pancreatic cancer cells, inducing apoptosis and showing synergy with gemcitabine [56].

In the present study, we have developed novel biocomposites using AmphPVP nano-sized particles encapsulating BTZ in the hydrophobic core and surface modified with either DR5-B or DR5-B-iRGD targeting ligands. BTZ was previously shown to be ineffective for pancreatic cancer treatment in Phase II study with panobinostat [57], which can be attributed to its low bioavailability. Similarly, the effect of antitumor cytokine TRAIL against pancreatic cancer was controversial [58], probably due to the affinity of wild type TRAIL to five receptors, including decoys DcR1, DcR2, and OPG. However, receptor-selective DR5-targeted formulations recently showed high antitumor effect in pancreatic cancer models [56,59]. The iRGD peptide is widely applied to enhance tissue penetration of various drugs [60]. Therefore, the biocomposite developed in current study combines several advantages, including the synergistic antitumor effect of DR5-selective variant DR5-B with BTZ [61], sustained BTZ release, as well as enhanced internalization of AmphPVP-BTZ-DR5-B-iRGD biocomposites due to the modification with tumor-penetrating fusion protein DR5-B-iRGD.

In vitro drug release demonstrates that the developed smart nano-scaled carrier system based on amphiphilic PVP exhibits a noticeable pH-responsive release of BTZ drug from the core of the particles. The release profiles and kinetic analysis provide clear insights into the underlying BTZ drug release mechanisms. The most interesting finding is the pronounced pH-dependent release dependence: pH 5.6 > pH 7.4 > pH 8.2. This trend can be directly connected with the physicochemical properties of the amphiphilic PVP polymer. The observed pH-dependent release is attributed to the properties of the amphiphilic PVP carrier, not BTZ itself, as the free drug diffuses rapidly without pH sensitivity [62]. We propose that in an acidic environment, protonation of amine groups (or other weakly basic fragments) occurs in the hydrophobic block of the AmphPVP. This increases the hydrophilicity of the nanoparticles’ polymer shell, leading to substantial swelling, enhanced polymer chain mobility, and a decrease in the particles’ thermodynamic stability, which facilitates more rapid BTZ diffusion. At neutral and alkaline pH, the core remains compact, restricting drug release.

The accelerated release in an acidic environment can be an additional feature of the combined effective tumor-targeting delivery system proposed. Upon local intratumoral administration, the biocomposites would form a drug depot with minimal initial burst release in the extracellular tumor environment (pH ~ 6.5–7.2). This helps retain the payload at the tumor site. Following cellular uptake by cancer cells via endocytosis, the nanoparticles are transported to endosomes (pH ~ 6.0–6.5) and finally lysosomes (pH ~ 4.5–5.0). The sharp increase in release rate at pH 5.6 demonstrates the system’s ability to rapidly unload its cytotoxic cargo specifically within these acidic compartments, leading to high intracellular drug concentrations and enhanced anticancer efficacy while minimizing systemic exposure.

The kinetic model analysis confirmed that the BTZ drug release mechanism from the Amp-PVP nanoparticles is complex and best described by the Korsmeyer–Peppas model. The poorest fit of zero-order model (R^2^ < 0.9) indicates that the release rate is not constant over time, which is typical for most polymeric drug delivery systems. The high R^2^ values (R^2^ > 0.98) for the Higuchi model suggest that diffusion plays a significant role throughout the process.

The kinetic analysis using the Korsmeyer–Peppas model offers a mechanistic explanation for this behavior. The release exponent (n) values indicate that the drug release follows an anomalous (non-Fickian) transport mechanism at all pH levels. This means release is governed by a combination of drug diffusion through the polymer matrix and polymer chain relaxation/swelling. The key results come from the trend in the *n* values. At pH 5.6, the highest n value (0.67) suggests a significant contribution from polymer relaxation. We propose that in an acidic environment, protonation of amine groups (or other weakly basic fragments) occurs in the hydrophobic block of the amphiphilic PVP. This increases the hydrophilicity of the nanoparticles’ polymer shell, leading to substantial swelling, enhanced polymer chain mobility, and a decrease in the particles’ thermodynamic stability which facilitates more rapid BTZ drug diffusion.

As the pH increases to 7.4 and then to 8.2, the n value decreases (0.58 and 0.52, respectively). This indicates a gradual shift in the release mechanism. In these conditions, the polymer hydrophobic fragments remain deprotonated and less hydrated. The particles’ core remains more compact and stable, also restricting PVP chain mobility. Consequently, the release becomes more diffusion-dominated, as evidenced by the lower n values and the excellent fit to the Higuchi model (R^2^ > 0.99). The slower diffusion through the dense polymer shell explains the progressively reduced release rates.

The inclusion of the alkaline condition (pH 8.2) was crucial, as it conclusively shows that the nano-scaled carriers are most stable and retentive in a non-acidic environment. This not only strengthens the pH-triggering idea but also suggests potential benefits for the formulation shelf-life, as drug leakage during storage would be minimized. Generally, the provided kinetic analysis confirms the pH-responsive “smart” nature of the developed nanocarrier.

This study serves as a robust in vitro proof-of-concept, demonstrating the feasibility and potential of the developed biocomposites. The logical next step, which is beyond the scope of this current work, involves comprehensive in vivo validation to assess pharmacokinetics, biodistribution, and safety profiles in animal models of pancreatic cancer.

While the large hydrodynamic diameter (~680 nm) and polydispersity (PDI = 0.5) of the developed core–shell biocomposites would hinder efficient extravasation and systemic circulation required for intravenous targeted delivery [63,64], these very characteristics position them as highly promising candidates for localized intratumoral (IT) administration. The reason for this application is multi-faceted and directly addresses the limitations of systemic chemotherapeutic delivery. Firstly, the submicron size of the biocomposites is ideal for creating a sustained-release drug depot within the tumor mass. It is known that particles in hundreds of nanometers to micrometer range exhibit significantly prolonged local retention compared to small-molecule drugs or smaller nanoparticles, which rapidly diffuse away via the systemic circulation or lymphatic drainage [65,66].

The immobilization of the DR5-B or DR5-B-iRGD protein on the particle surface via non-covalent bonding with the PVP corona increases the protein valency. This multivalent presentation is known to enhance death receptor clustering and amplify apoptotic signaling compared to soluble ligand [67]. This mechanism is supported by our finding that DR5 receptor internalization occurs more rapidly upon exposure to AmphPVP-BTZ-DR5-B and AmphPVP-BTZ-DR5-B-iRGD rather than to free proteins At the same time, sustained release of the proteasome inhibitor bortezomib (BTZ) from the hydrophobic core can sensitize cancer cells to DR5-mediated apoptosis, creating a powerful, localized combinatorial therapy that minimizes systemic exposure and off-target side toxicity [68]. Importantly, our results show that the substantial enhancement of cytotoxicity of AmphPVP BTZ DR5 B and PVP BTZ DR5 B-iRGD biocomposites compared to AmphPVP BTZ can be attributed not only to the synergistic action of the antitumor agents DR5-B and DR5-B-iRGD with BTZ. Since modification with DR5-B or DR5-B-iRGD enhances DR5 receptor-mediated internalization of the PVP BTZ DR5 B or PVP BTZ DR5 B-iRGD biocomposites into cells, it also may enable the accelerated release of bortezomib in acidic intracellular environment compared to PVP BTZ.

The physicochemical properties of the prepared biocomposites further support their use for IT injection. The near-neutral zeta potential (−4 mV) suggests limited non-specific interaction with negatively charged components of the extracellular matrix (ECM), which may help a more uniform distribution of the depot from the injection site [69]. Furthermore, while a PDI of 0.5 indicates a moderate degree of heterogeneity, this is less crucial for IT administration where the primary requirement is that the entire particle distribution is within a size range that ensures local retention, which is comfortably met by obtained submicron polymeric biocomposites.

## 4. Materials and Methods

### 4.1. Materials

Amphiphilic polymer of N-vinylpyrrolidone with one terminal n-octadecyl hydrophobic fragment and molecular weight of 11 kDa (D.I. Mendeleev University of Chemical Technology of Russia, Moscow, Russia); bortezomib (Santa Cruz Biotechnology, Inc, Dallas, TX, USA); chloroform and dimethylsulfoxide (DMSO) (ChimMed, Moscow, Russia); Dulbecco’s modified Eagle’s Medium (DMEM); trypsin-versene solution and phosphate-salt tablets (PanEco, Moscow, Russia); fetal bovine serum (FBS) (Cytiva, Marlborough, MA, USA); 3-(4,5-dimethylthiazol-2-yl)-2,5-diphenyl-tetrazolium bromide (MTT) reagent (Millipore-Sigma, St. Louis, MO, USA). All solvents and components of buffer solutions were of analytical grade and used as received.

### 4.2. Expression and Purification of TRAIL DR5-B and DR5-B-iRGD

TRAIL DR5-B and DR5-B-iRGD were obtained as a recombinant protein by heterologous expression in bacterial cells as previously described [70]. Briefly, plasmids pET32a/dr5-b or pET32a/dr5-b-irgd were transformed in *E.coli* SHuffle B strain. Proteins were expressed at 28 °C for 20 h and then cells were destroyed under a pressure of 2000 psi using a French Press (Spectronic Instruments, Inc., Rochester, NY, USA). The proteins were purified from a soluble fraction of cytoplasmic proteins consequently by immobilized metal affinity chromatography on Ni-NTA agarose (Qiagen GmbH, Germantown, MD, USA) and ion-exchange chromatography on SP Sepharose (GE Healthcare, Danderyd, Sweden). The protein solutions were dialyzed against 150 mM NaCl, 50 mM NaH_2_PO_4_, pH 7.5 for 24 h at +4 °C, sterilized by filtration, lyophilized, and stored at −20 °C. The purity was confirmed using polyacrylamide gel electrophoresis.

### 4.3. Fabrication Nanoparticles AmphPVP-BTZ

AmphPVP-BTZ particles were obtained from the amphiphilic polymer N-vinylpyrrolidone by solvent evaporation method [43]. Briefly, polymer and bortezomib (2% mass from polymer) were dissolved in chloroform and mixed with water then homogenized by ultrasound (Bandelin Sonopuls HD 4400; Bandelin GmbH & Co. KG, Berlin, Germany), and chloroform was removed using a rotor evaporator (IKA RV10 basic; IKA, Staffen im Breisgau, Germany). Non-encapsulated BTZ was separated from the particle suspension by centrifugation for 5 min at 4000 *g* (Sigma 4-5L centrifuge; Merck KGaA, St. Louis, MO, USA). The supernatant was frozen and freeze-dried (BETA 2 2-8 LSCplus lyophilizer; Martin Christ, Gefriertrocknungsanlagen GmbH, Osterode am Harz, Germany). The loading efficiency and loading capacity of BTZ in particles were determined spectrophotometrically at 270 nm (UV-Vis spectrophotometer 1900i; Shimadzu Corporation, Kioto, Japan).

### 4.4. Fabrication Nanocomposites AmphPVP-BTZ-DR5-B and AmphPVP-BTZ-DR5-B-iRGD

AmphPVP-BTZ-DR5-B and AmphPVP-BTZ-DR5-B-iRGD were obtained by incubating AmphPVP-BTZ with DR5-B or DR5-B-iRGD in phosphate-buffered saline (pH 7.0) for 12 h. The suspensions were centrifuged with 10,000 *g* for 10 min, the supernatant was removed, and the precipitate was resuspended in phosphate-buffered saline (pH 7.0). The concentration of DR5-B or DR5-B-iRGD was measured by Bradford using polymeric particles non-modified with protein as control samples. The sorption capacity was determined by the amount of protein from 1 mg of AmphPVP-BTZ particles.

### 4.5. Transmission Electron Microscopy (TEM)

TEM measurements were carried out using a JEM-1400 (JEOL Ltd., Tokyo, Japan) microscope operating at 120 kV. The formvar/carbon TEM grids (TedPella, Redding, CA, USA) were treated using a glow-discharge device Emitech K100X (Quorum Technologies Ltd., Laughton, UK) at 25 mA current, the treatment time was 45 s. The samples were deposited onto the grid surface for 1 min, then the grids were blotted and stained by 1% uranyl acetate twice. The images were stored as tiff files and processed using Fiji software (ver 1.54p) [71].

### 4.6. Dynamic Light Scattering (DLS)

The average hydrodynamic diameter and polydispersity index were determined by dynamic light scattering, and the ζ-potential was determined by electrophoretic light scattering using the Zetasizer Nano ZS (Malvern Instruments, Ltd., Worcestershire, UK). The measurements were carried out three times at 25 °C.

### 4.7. In Vitro Drug Release Study

The release profile of DR5-B or DR5-B-iRGD protein from AmphPVP-BTZ-DR5-B and AmphPVP-BTZ-DR5-B-iRGD nanocomposites was stored in 150 mM NaCl, 1 mM NaH_2_PO_4_ (pH 7.0) at +4 °C. In a certain period of time (1, 2, 3, 4, 6 weeks), nanocomposites were centrifuged at 10,000× *g* for 10 min, and the protein contents in the supernatants and in the resuspended nanoparticle pellets were measured by Bradford assay.

The release profile of BTZ from AmphPVP-BTZ nanoparticles was investigated using a dialysis method under sink conditions. The study was conducted at 37 °C under stirred conditions (200 ± 50 rpm) to simulate physiological temperature and agitation. To simulate different biological environments, three release media were used. PBS with pH 5.6 was used to mimic the acidic environment of endosomes/lysosomes. To simulate the physiological pH of the blood, PBS was used with pH 7.4. Finally, Tris-HCl buffer with pH 8.2 was utilized to probe polymeric carrier behavior in a slightly alkaline environment.

Briefly, 10 mL of the AmphPVP-BTZ nanoparticles was placed in a dialysis bag (molecular weight cut-off (MWCO) 6 kDa, Thermo Fisher Scientific, Inc., Waltham, MA, USA). This bag was immersed in 100 mL of the respective release medium (pH 5.6, 7.4, or 8.2). At predetermined time intervals (0, 0.5, 1, 2, 4, 6, 8, 12, 24, and 48 h), 1 mL aliquots were withdrawn from the external release medium. An equal volume of fresh, pre-warmed buffer was immediately added back to maintain a constant volume. The concentration of BTZ in the withdrawn samples was quantified using a UV-Vis spectrophotometer (RF-6000, Shimadzu Corporation, Kioto, Japan) at a wavelength of 270 nm. The cumulative percentage of BTZ released was calculated and plotted against time. All experiments were performed in triplicate.

The release data were fitted to various mathematical models to investigate the release mechanism. In this work, the release curves of the BTZ from Amph-PVP NPs were fitted using zero-order release kinetics (*Q_t_* = *Q*_0_−*K*_0_*t*), first-order kinetics model (*logQ_t_* = *logQ*_0_ − (*K*_1_*t*)/2.303), the Higuchi model (*Q_t_* = *K_H_t*^0.5^), and the Korsmeyer–Peppas model (*Q_t_* = *Kt^n^*), where Q_t_—cumulative amount of drug released at time t; Q_0_—initial drug amount in the matrix; *K*_0_—zero-order release rate constant; *K*_1_—first-order release rate constant; *K*_H_—Higuchi’s release rate constant; *K*—Korsmeyer–Peppas rate constant; *n*—release exponent relating to transport mechanism. These particular models are usually used to describe drug release from polymeric systems and to predict the nature of the temporary drug release [72,73,74,75]. The goodness of fit of the models was assessed by the correlation coefficient (R^2^).

### 4.8. Cell Lines

Pancreatic adenocarcinoma cell lines MIA PaCa-2 (Cat. No. CRL-1420), PANC-1 (Cat. No. CRL-1469), BxPC-3 (Cat. No. CRL-1687), and human epithelial fibroblasts (Cat. No. PCS-201-012) were obtained from the American Collection of Type Cell Cultures (Manassas, VA, USA). The cells were cultured in a DMEM containing 5% fetal bovine serum and 100 micrograms/mL of penicillin and streptomycin, at 37 °C and 5% CO_2_. The cells were detached with 0.25% trypsin-EDTA and transplanted when the density reached 70–80%.

### 4.9. Cytotoxicity Assay

The cytotoxicity of AmphPVP-BTZ-DR5-B and AmphPVP-BTZ-DR5-B-iRGD nanocomposites was studied using MTT assay. The cells were seeded into a 96-well plate (7 × 10^3^ cells/well) and incubated for 24 h at 37 °C and 5% CO_2_. BTZ was dissolved in DMSO at a concentration of 10 mM and added to the cells so that final DMSO content in the well did not exceed 0.5% and did not exhibit a cytotoxic effect. DMSO effects were controlled by the vehicle. DR5-B, DR5-B-iRGD, AmphPVP-BTZ, AmphPVP-BTZ-DR5-B, or AmphPVP-BTZ-DR5-B-iRGD were added in different concentrations and incubated for 24 h. Then 0.05% *(w*/*v)* MTT reagent was added to each well (final concentration 0.5 mg/mL) and incubated for 4 h. The formazan crystals were dissolved in DMSO and the optical density was measured spectrophotometrically at a wavelength of 595 nm and a reference wavelength of 655 nm using the iMark plate spectrophotometer (Bio-Rad, Hercules, CA USA) [76]. Cell viability (%) was calculated using the following formula: (optical density of the sample—optical density of the background)/(optical density of the control − optical density of the background) × 100%. The concentration of semi-maximal inhibition (IC_50_) was calculated in GraphPad Prism 8.0 (GraphPad Software Inc., San Diego, CA, USA) according to the built-in formula.

### 4.10. Western Blotting

MIA PaCa-2 cells were seeded in 25 cm^2^ cell culture flasks (3 × 10^6^ cells/flask) for 24 h, treated with 50 nM of ligands or biocomposites for 4 h, and lysed in 300 µL of weak RIPA lysis buffer (Servicebio, Wuhan, China) supplemented with Halt Protease Inhibitor Single-Use Cocktail (Thermo Fisher Scientific, USA). Total protein concentration was determined by the Bradford assay, and 30 μg of protein per lane were subjected to 12% SDS–PAGE under reducing conditions in sample buffer containing β-mercaptoethanol. Proteins were transferred onto 0.45-μm PVDF membranes (Servicebio, China) using a wet transfer system (Bio-Rad, USA). Membranes were blocked in TBST buffer containing 5% (*w*/*v*) non-fat dry milk for 1 h at room temperature and incubated overnight at 4 °C with primary antibodies: anti-PARP mouse mAb (clone 123, Thermo Fisher Scientific; 1:1000, 0.5 μg/mL) or anti-caspase-3 rabbit pAb (GeneTex, Irvine, CA, USA; 1:1000, 1.3 μg/mL). After washing with TBST, membranes were incubated for 1 h at room temperature with HRP-conjugated secondary antibodies: goat anti-mouse IgG-HRP (Immun-Star, Bio-Rad; 1:2500, 0.32 μg/mL) or goat anti-rabbit IgG-HRP (Jackson ImmunoResearch, Philadelphia, PA, USA; 1:1000, 0.8 μg/mL). GAPDH was used as a loading control (rabbit anti-GAPDH mAb, clone 6C4F6, Servicebio; 1:2000, 0.3 μg/mL). Signals were developed using an ECL substrate (Bio-Rad) and acquired with a C-DiGit^®^ Blot Scanner (LI-COR, Lincoln, NE, USA).

### 4.11. Internalization of the DR5 Receptor by Flow Cytometry

MIA PaCa-2 cells were seeded in 6-well plates (5 × 10^5^ cells/well) at 37 °C, 5% CO_2_ for 48 h, and incubated with 5 nM of DR5-B, DR5-B-iRGD, AmphPVP-BTZ, AmphPVP-BTZ-DR5-B, or AmphPVP-BTZ-DR5-B-iRGD for 60, 30, 15, and 5 min. Furthermore, the cells were washed thrice with ice-cold PBS, detached by Versene solution, resuspended in FACS buffer (1% BSA in PBS), and incubated for 1 h at +4 °C with monoclonal antibodies to DR5 (clone DR5-01-1; GeneTex, Inc., Irvine, CA, USA). The cells were then washed thrice and incubated for 1 h at +4 °C with 1:100 of secondary antibodies (RepertoireLab, St.Petersburg, Russia). The cells were then washed and suspended in FACS buffer with propidium iodide. The DR5 surface expression was assessed using CytoFlex flow cytometer (Beckman Coulter, Inc., Indianapolis, IN, USA). Mouse IgG1 (clone 15H6; GeneTex Inc., Irvine, CA, USA) was used as an isotype control.

### 4.12. Surface Expression of the DR5 Receptor by Flow Cytometry

MIA PaCa-2, BxPC-3, PANC-1 cells were seeded in 6-well plates (4 × 10^5^ cells/well) at 37 °C, 5% CO_2_ for 48 h, and incubated with 5 nM (by DR5-B) tested samples for 15 and 5 min. Further, the cells were washed thrice with ice-cold PBS, detached by EDTA solution, suspended in FACS buffer (1% BSA in PBS), and incubated for 1 h at +4C with monoclonal antibodies to DR5 (clone DR5-01-1; GeneTex Inc., Irvine, CA, USA). The cells were then washed thrice and incubated for 1 h at +4C with secondary antibodies DyLight 488 (GeneTex Inc., Irvine, CA, USA). The cells were then washed and suspended in FACS buffer with propidium iodide. The mouse IgG1 (isotype control) was stained in parallel. The detection was performed on a CytoFLEX cytometer (Beckman Coulter, Indianapolis, IN, USA), collecting at least 10,000 live cell events. The level of DR5 was assessed by the median fluorescence (DMFI) relative to the isotypic control.

### 4.13. Study of Mechanism of Cell Death

MIA PaCa-2 cells were seeded into a 96-well plate (7 × 10^3^ cells/well) and incubated for 24 h at 37 °C and 5% CO_2_. The pan-caspase inhibitor Z-Vad(OMe)-FMK was dissolved in DMSO at a concentration of 10 mM and added to wells at a concentration of 20 µM. DMSO with a 1% concentration was added to the control wells and incubated for 1 h. 50 nM DR5-B, DR5-B-iRGD, AmphPVP-BTZ, AmphPVP-BTZ-DR5-B, or AmphPVP-BTZ-DR5-B-iRGD were added and incubated for 24 h. Then 0.05% MTT reagent was added to each well and incubated for 4 h. The formed crystals were dissolved in DMSO, and the optical density was measured spectrophotometrically at a wavelength of 595 nm and a reference wavelength of 655 nm. Cell viability (%) was calculated using the following formula: (optical density of the sample − optical density of the background)/(optical density of the control is the optical density of the background) ×100%.

### 4.14. Statistical Analysis

All measurements were performed at least three times and presented as means ± SD. The data was analyzed using one-way ANOVA followed by Tukey’s multiple comparisons test, and *p* < 0.05 indicated significant difference between groups.

## 5. Conclusions

The developed AmphPVP biocomposites loaded with BTZ and modified with either DR5-B or DR5-B-iRGD targeted ligand represent a strategically designed and innovative platform for locoregional cancer therapy. The key novelty of this work lies in the synergistic combination of a proteasome inhibitor (BTZ) with a DR5-selective apoptotic ligand within a single, pH-responsive amphiphilic AmphPVP carrier, further enhanced by a tumor-penetrating peptide (iRGD). Their size can ensure prolonged tumor residence, their core–shell structure enables a synergistic multi-modal mechanism of action, and their surface chemistry is suited for local deployment. Their smart pH-responsive behavior ensures selective BTZ release in the acidic compartments of cancer cells, which is a key strategy for improving therapeutic outcomes while minimizing systemic drug exposure and associated side effects. The well-defined release mechanism, transitioning from relaxation-erosion control at low pH to diffusion control at higher pH, provides a solid basis for the further development and optimization of this drug delivery system. High cytotoxicity on pancreatic adenocarcinoma cells without significant cytotoxicity on healthy cells makes the fabricated biocomposites promising for pancreatic cancer therapy. Future work will focus on in vivo evaluation of tumor retention and efficacy in relevant pancreatic cancer models. It is important to note the limitations of the present study, which are primarily based on in vitro proof-of-concept. Future investigations should include evaluation in 3D spheroid or organoid models that better mimic the tumor microenvironment, and comprehensive in vivo pharmacokinetic and toxicological profiling. Regarding scale-up, the fabrication process utilizes relatively simple and scalable techniques like solvent evaporation and physical adsorption, which is promising for future translational development. However, optimizing the process to achieve a narrower particle size distribution and ensuring long-term stability of the lyophilized formulations will be critical steps for successful industrial translation.

## Figures and Tables

**Table 1:** The main characteristics of AmphPVP, AmphPVP-BTZ, AmphPVP-BTZ-DR5-B, and AmphPVP-BTZ-DR5-B-iRGD biocomposites.

	Z-Average Size, (nm ± SD)	Zeta Potential, (mV ± SD)	PDI ^1^	Sorption Capacity, µg Protein/mg AmphPVP-BTZ
AmphPVP	180 ± 12	−8.9 ± 2.1	0.16	-
AmphPVP-BTZ	300 ± 16	−6.7 ± 1.6	0.23	-
AmphPVP-BTZ-DR5-B	680 ± 20	−4.2 ± 2.6	0.48	5.0 ± 0.5
AmphPVP-BTZ-DR5-B-iRGD	684 ± 15	−3.8 ± 2.9	0.52	4.5 ± 0.5

^1^ Polydispersity index.

**Table 2:** Kinetic model parameters for BTZ release from the biocomposites.

Model/Parameter	pH 5.6	pH 7.4	pH 8.2
Zero-Order (R^2^)	0.837	0.876	0.894
First-Order (R^2^)	0.946	0.967	0.975
Higuchi (R^2^)	0.983	0.991	0.993
Korsmeyer-Peppas			
n (release exponent)	0.67	0.58	0.52
K (rate constant, h^−n^)	18.45	15.12	13.88
**R^2^**	0.999	0.998	0.998

**Table 3:** Half maximal inhibitory concentrations (IC_50_) of DR5-B, DR5-B-iRGD, AmphPVP, AmphPVP-BTZ, AmphPVP-BTZ-DR5-B, or AmphPVP-BTZ-DR5-B-iRGD in pancreatic cancer cell lines.

	IC_50_, nM		
	PANC-1	BxPC-3	MIA PaCa-2
DR5-B	2.35 ± 1.47	0.35 ± 0.11	0.17 ± 0.02
DR5-B-iRGD	0.18 ± 0.04	0.09 ± 0.01	0.07 ± 0.01
AmphPVP-BTZ	35.03 ± 5.24	56.22 ± 5.09	34.01 ± 3.50
AmphPVP-BTZ-DR5-B	0.13 ± 0.03 *	0.007 ± 0.001 *	0.006 ± 0.001 *
AmphPVP-BTZ-DR5-B-iRGD	0.059 ± 0.011 ^#^	0.003 ± 0.001 ^#^	0.001 ± 0.001 ^#^

* Significant difference from AmphPVP-BTZ and DR5-B; **^#^** Significant difference from AmphPVP-BTZ-DR5-B. One-way ANOVA with Tukey’s multiple comparisons test, *p* < 0.0001.

## Data Availability

The original contributions presented in this study are included in the article/Supplementary Materials. Further inquiries can be directed to the corresponding author.

## Supplementary Materials

The following supporting information can be downloaded at https://www.mdpi.com/article/10.3390/ijms262311620/s1.

## Abbreviations

The following abbreviations are used in this manuscript:

BTZ	Bortezomib
PDI	Polydispersity index
TEM	Transmission electron microscopy
DLS	Dynamic light scattering
MTT	3-(4,5-dimethylthiazol-2-yl)-2,5-diphenyl-tetrazolium bromide
DMEM	Dulbecco’s modified Eagle’s Medium
DMSO	Dimethylsulfoxide
FBS	Fetal bovine serum
IT	Intratumoral
