# Supplementary material for: Amphiphilic Poly(N-vinylpyrrolidone) Biocomposites with Bortezomib and DR5-Selective TRAIL Variants: A Promising Approach to Pancreatic Cancer Treatment
Source: Int J Mol Sci. 2025 Nov 30;26(23):11620. doi: 10.3390/ijms262311620 (PMC12691830; doi:10.3390/ijms262311620)
Supplement: Supplementary file 1 [file ijms-26-11620-s001.zip › ijms-3964107-supplementary.pdf]

Supplementary material

Amphiphilic poly(N-vinylpyrrolidone) Biocomposites with Bortezomib and DR5-Selective TRAIL Variants: A Promising Approach to Pancreatic Cancer Treatment

Ekaterina Kukovyakina[1], Alina Isakova[1,2,3], Dmitry Bagrov[2], Marine Gasparian[3], Andrey Kuskov[1#] and Anne Yagolovich[2*#]

[1] Department of Technology of Chemical, Pharmaceutical and Cosmetic Substances, D. Mendeleev University of Chemical Technology of Russia, 125047 Moscow, Russia; kukoviakina.e.v@muctr.ru, kuskov.a.n@muctr.ru
[2] Faculty of Biology, Lomonosov Moscow State University, 119234 Moscow, Russia; alina.labbio@gmail.com, bagrov@mail.bio.msu.ru, yagolovichav@my.msu.ru
[3] Shemyakin-Ovchinnikov Institute of Bioorganic Chemistry of the Russian Academy of Sciences,
117997 Moscow, Russia; marine_gasparian@yahoo.com,
* Correspondence yagolovichav@my.msu.ru
# These authors have contributed equally to the work.

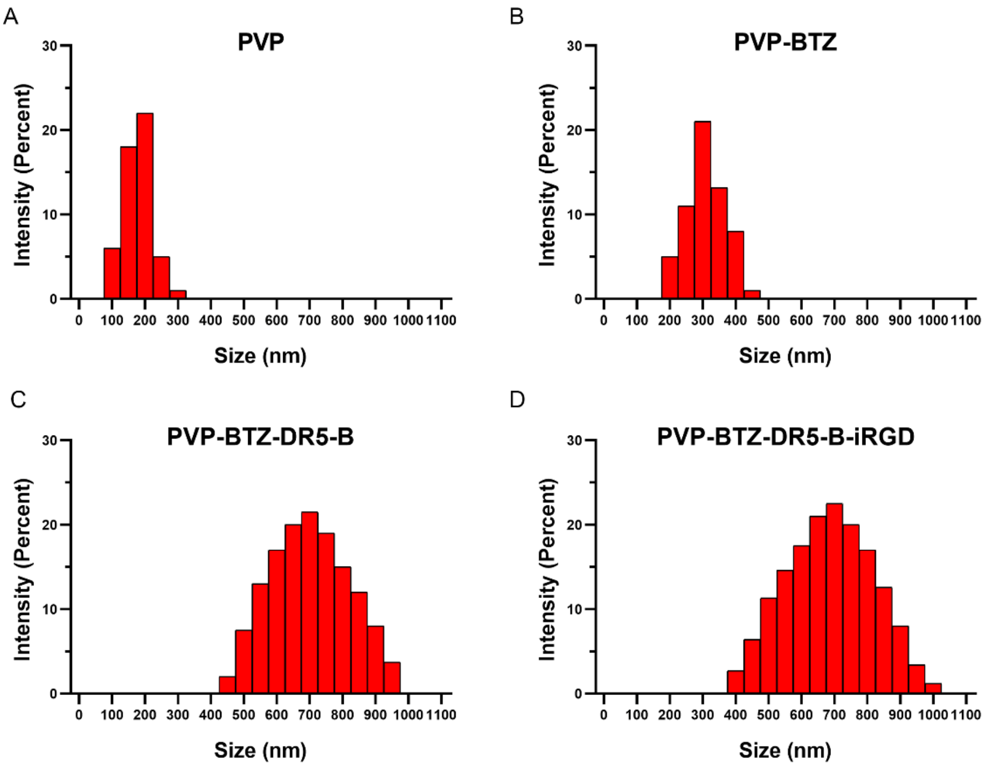

Figure S1. DLS size distribution plots: (**a**) AmphPVP; (**b**) AmphPVP-BTZ; (**c**) AmphPVP-BTZ-DR5-B; (**d**) AmphPVP-BTZ-DR5-B-iRGD. All data were expressed as means ± SD (n=3).

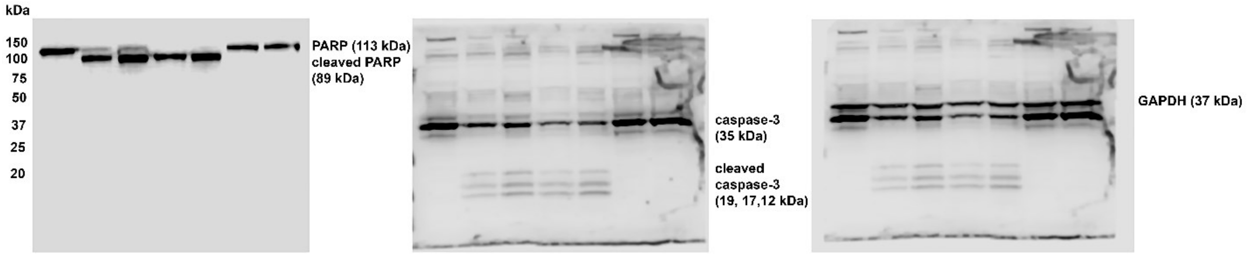

Figure S2. Western blot analysis of PARP and caspase-3 cleavage in MIA PaCa-2 cells induced by 50 nM of ligands after 4 h incubation. Staining with monoclonal antibodies to PARP1 (Invitrogen, clone 123) 1:500, Thermo Fisher Scientific, USA; polyclonal antibodies to caspase-3 (Cat. No. GTX110543), 1:1000, GeneTex, USA; monoclonal antibodies to GAPDH (clone 6C4F6), 1:2000, Servicebio, China.
